# Ageing related thyroid deficiency increases brain-targeted transport of liver-derived ApoE4-laden exosomes leading to cognitive impairment

**DOI:** 10.1038/s41419-022-04858-x

**Published:** 2022-04-25

**Authors:** Manman Zhang, Wenliang Gong, Dianjun Zhang, Ming Ji, Binjie Chen, Beina Chen, Xinyu Li, Yuefei Zhou, Chengyi Dong, Gehua Wen, Xiaoni Zhan, Xiafang Wu, Lulu Cui, Yuliang Feng, Siman Wang, Huiya Yuan, Enyu Xu, Maosheng Xia, Alexei Verkhratsky, Baoman Li

**Affiliations:** 1 Department of Forensic Analytical Toxicology, School of Forensic Medicine, China Medical University, Shenyang, China; 2 Department of Orthopaedics, The First Hospital, China Medical University, Shenyang, People’s Republic of China; 3 Faculty of Biology, Medicine and Health, The University of Manchester, Manchester, UK; 4 Achucarro Center for Neuroscience, IKERBASQUE, 48011 Bilbao, Spain; 5 Department of Stem Cell Biology, State Research Institute Centre for Innovative Medicine, LT-01102 Vilnius, Lithuania

**Keywords:** Cognitive neuroscience, Alzheimer's disease, Cellular neuroscience

## Abstract

Alzheimer’s disease (AD) is the prevalent cause of dementia in the ageing world population. Apolipoprotein E4 (ApoE4) allele is the key genetic risk factor for AD, although the mechanisms linking ApoE4 with neurocognitive impairments and aberrant metabolism remains to be fully characterised. We discovered a significant increase in the ApoE4 content of serum exosomes in old healthy subjects and AD patients carrying ApoE4 allele as compared with healthy adults. Elevated exosomal ApoE4 demonstrated significant inverse correlation with serum level of thyroid hormones and cognitive function. We analysed effects of ApoE4-containing peripheral exosomes on neural cells and neurological outputs in aged or thyroidectomised young mice. Ageing-associated hypothyroidism as well as acute thyroidectomy augmented transport of liver-derived ApoE4 reach exosomes into the brain, where ApoE4 activated nucleotide-binding oligomerisation domain-like receptor family pyrin domain-containing 3 (NLRP3) inflammasome by increasing cholesterol level in neural cells. This, in turn, affected cognition, locomotion and mood. Our study reveals pathological potential of exosomes-mediated relocation of ApoE4 from the periphery to the brain, this process can represent potential therapeutic target.

## Introduction

Alzheimer’s disease (AD) is the leading cause of age-associated neurodegeneration, with no effective therapeutic management. Apolipoprotein E (ApoE) serves multiple functions including maintenance of the structure of the lipoprotein particles and regulation of the metabolism of several lipoproteins [1]. Human ApoE exists in three isoforms, ApoE2, ApoE3, and ApoE4, which differ only in two residues. The ApoE4 allele has been reported as the genetic risk factor for late-onset, sporadic AD almost three decades ago [2–4], yet how ApoE4 predisposes to AD remains incompletely understood. Several reports identified an association between dysregulation of thyroid hormones (THs) and AD [5–7]. Serum levels of THs (total triiodothyronine (TT3), free T3 (FT3), total thyroxine (TT4) and free thyroxine (FT4)) gradually decrease with age in humans and rodents [8–10]. Whether thyroid hormonal status is affected in ApoE4 allele carriers is unknown. Systemic ApoE4 is mainly produced by hepatocytes, whereas in the brain ApoE4 is primarily expressed in and secreted by astrocytes [11, 12]. The 34 kDa ApoE4 cannot pass the blood–brain barrier (BBB). This transport, however, can be mediated by exosomes, which were reported to carry nucleic acid and proteins across BBB [13]. The existence of ApoE in the human plasma exosomes has been also reported recently [14]. Vacuolar ATPases (V-ATPases) are ATP-dependent proton pumps, highly expressed in the ruffled border membranes to acidify the resorption lacunae [15]. In the liver, V-ATPases are involved in the formation of lysosomes [16], in tissue regeneration and tumorigenesis [17]. Mutations in ATP6AP1 subunit of V-ATPase lead to liver malfunction [18]. It remains unknown whether THs regulate the function of V-ATPase in liver [15]. In this study, we demonstrate that age-dependent decline in THs accelerates exosomal delivery of liver-derived ApoE4 into the brain by regulating the expression of ATPase H^+^ Transporting Accessory Protein 1 (ATP6AP1). Accumulation of peripheral ApoE4 in the brain in turn affects neural cells and instigates cognitive impairments.

## Results

### Multiple correlations between THs level, cognitive abilities and ApoE4 -containing serum exosomes in cognitively preserved and cognitively compromised human subjects

**Fig. 1:**
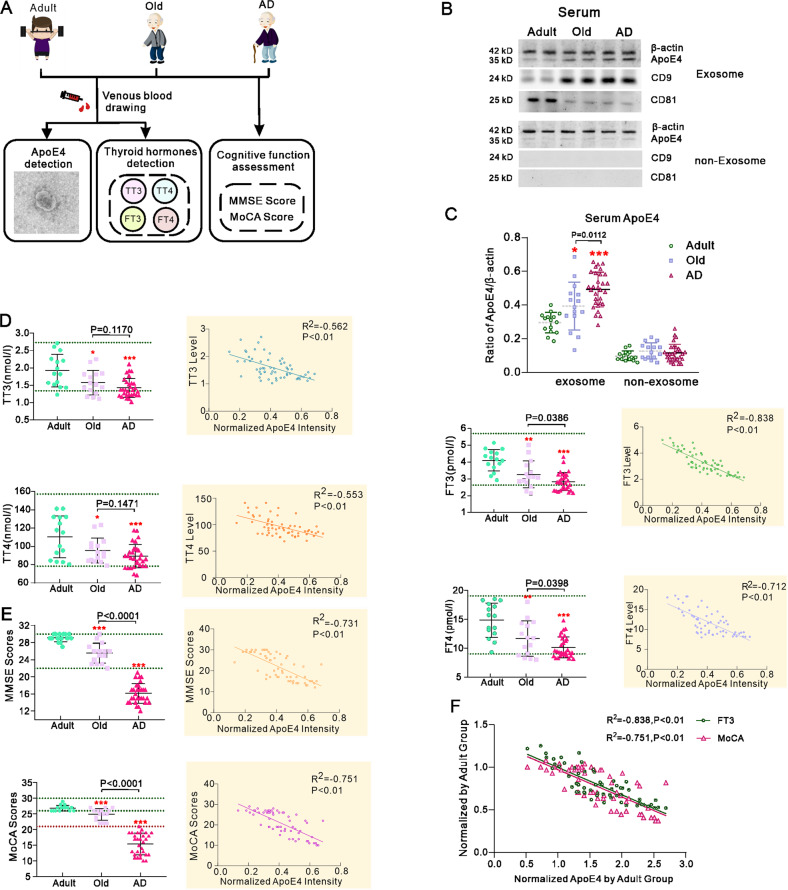
Correlation of ApoE4 level in serum exosomes with thyroid hormones levels and cognitive performance in human subjects. **A** Enroled 60 carriers of ApoE4 allele included 15 adult healthy subjects, 15 old healthy subjects and 30 old AD patients. Collected serum was used to extract exosomes and measure TT3, FT3, TT4 and FT4. Participants cognitive abilities were assessed by MMSE and MoCA scoring. **B** Representative western blots of ApoE4 and exosomal specific marker CD81 and CD9 in the serum extracted exosomes and the supernatant from which exosomes precipitate was removed after ultracentrifuge. **C** Normalised intensities of ApoE4 by β-actin. **D** TT3, FT3, TT4 and FT4 in adult, old and AD groups. Correlation analyses between the ApoE4 level in serum exosomes and TH levels are shown in inserts at the right. **E** MMSE and MoCA scores; correlation analyses between the ApoE4 level in serum exosomes and these scores are shown in inserts at the right. **F** Multiple correlation analysis of the normalised ApoE4 in serum exosomes with the normalised MoCA scores and serum FT3 in adult group. The individual measured value was plotted and shown as mean ± SD. One-way ANOVA for comparisons including more than two groups; unpaired two-tailed *t*-test for two-group comparisons. *p < 0.05, **p < 0.01, ***p < 0.001.

**Fig. 2:**
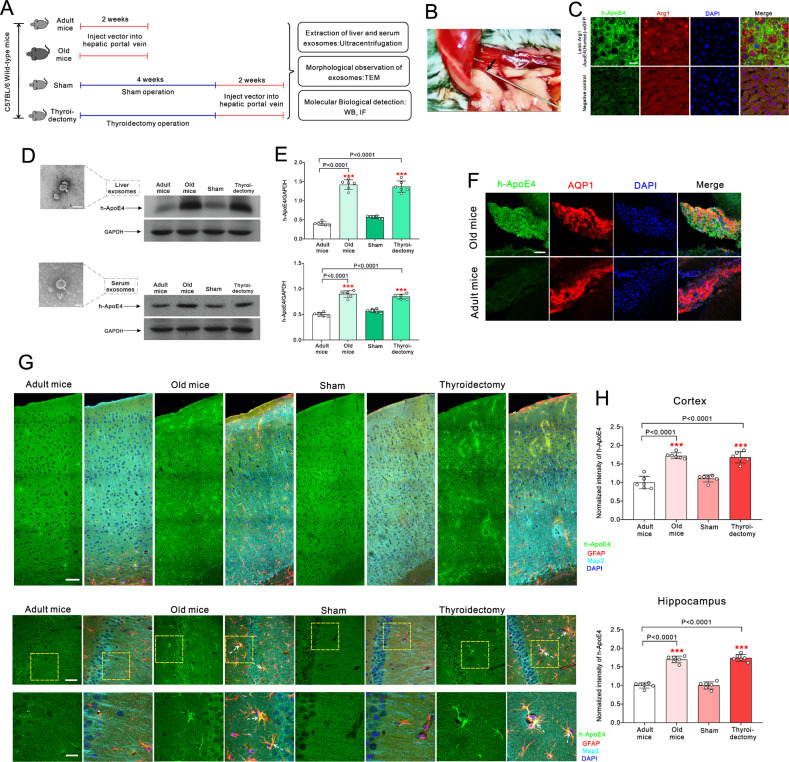
Ageing and thyroidectomy increased exosomal transport of liver-derived ApoE4 into the brain. **A** Experimental design: adult and old mice were injected with lenti-Arg1-ApoE4-eGFP vector through hepatic portal veins; 2 months-old mice were randomly operated sham or thyroidectomy. Four weeks after surgery mice were injected lenti-Arg1-ApoE4-eGFP vector through hepatic portal veins. **B** Injection of lentivirus vectors of h-ApoE4 and Arg1 (Lenti-Arg1-ApoE4-eGFP) through hepatic portal vein. **C** Immunofluoresence images taken from liver tissue of h-ApoE4 (green) and Arg1 (red) with nucleus marker DAPI (blue) performed 3 days after injection of lenti-Arg1-ApoE4-eGFP or lentivirus negative control vectors in adult mice. Scale bar = 50 μm. **D** Electron microscope images of exosome extracted from liver and serum (left), and the representative Western blots for h-ApoE4 in exosomes extracted from liver and blood serum (right). **E** Relative expression ratio of h-ApoE4 and GAPDH is plotted as mean ± SD, *n* = 6. One-way ANOVA for comparisons including more than two groups; unpaired two-tailed *t*-test for two-group comparisons. Compared to adult group, *p < 0.05, **p < 0.01, ***p < 0.001. **F** Immunofluorescence images of h-ApoE4 (green) co-stained with AQP1 (red) and DAP1 (blue) in choroid plexus. Scale bar = 50 μm. **G** Immunofluorescence images of h-ApoE4 (green) co-stained with AQP1 (red) and DAP1 (blue) in the whole cortex and hippocampus. Scale bar = 50 μm. **H** Immunofluorescence intensity of h-ApoE4 normalised to adult group. The normalised intensity is plotted as mean ± SD, *n* = 6. One-way ANOVA for comparisons including more than two groups; unpaired two-tailed *t*-test for two-group comparisons. One-way ANOVA for comparisons including more than two groups; unpaired two-tailed *t*-test for twogroup comparisons. Compared to adult group, *p < 0.05, **p < 0.01, ***p < 0.001.

We selected 60 ApoE4 heterozygous subjects including 15 healthy adults (22–48 years old), 15 healthy elderly (68–85 years old) and 30 AD patients (69–83 years old). All participants were subjected to cognitive evaluation with mini-mental state examination (MMSE); their cognitive status was correlated with blood levels of THs including TT3, FT3, TT4 and FT4, and ApoE4 content of serum exosomes (Fig. 1A). The level of ApoE4 normalised to β-actin in serum exosomes was significantly increased in old and AD groups compared with adult group (Fig. 1B), whereas ApoE4 content of serum exosomes in the AD group was significantly higher than in the old healthy subjects (*p* = 0.0112). At the same time, ApoE4 levels did not differ between adult, old and AD subjects in the serum supernatant after exosomes removal by ultracentrifugation (Fig. 1C). Exosomes were round, phospholipid bilayer‑enclosed structures with a diameter of 40‑100 nm as revealed by transmission electron microscopy and nanoparticle tracking analysis (Fig. 2D and Supplementary Fig. 1). Expression of the exosomal markers CD81 and CD9 was also tested in the extracted exosomes and the non-exosomal membranes. Serum levels of TT3, FT3, TT4 and FT4 decreased in old and AD groups when compared to young adults, although there was no significant difference between old and AD subjects (Fig. 1D). All THs were inversely linearly correlated to the normalised ApoE4/β-actin level (*p* < 0.01; Fig. 1D-right inserts). In the cognitive related tests, the MMSE and Montreal Cognitive Assessment (MoCA) scores were significantly decreased in AD group as compared with young and old healthy subjects (Fig. 1E). Correlations between MMSE or MoCA scores and the corresponding ApoE4 exosomal content similarly presented inversed linear relationship (*p* < 0.01; Fig. 1E-right inserts). According to the value of R^2^, the most relevant indicators are FT3 levels and MoCA scores; the multiple correlation analysis demonstrated that exosomal ApoE4 content presents significant inverse relationship with FT3 level and cognitive capacities (Fig. 1F). Increased exosomal ApoE4 was associated with the lower THs level and impaired cognition, hence we may suggest that age-dependent thyroid insufficiency promotes exosomal transportation of peripheral ApoE4 into the brain thus inducing cognitive decline.

### Ageing and hypothyroidism accelerated ApoE4 transport from the liver to the brain

To identify changes of exosomal ApoE4 transport with ageing, we studied adult (3 months-old) and old (18 months-old) wild type mice, while 2 months-old mice were randomly separated into sham and thyroidectomy surgery groups. The experimental design is shown in Fig. 2A.

Lentiviral transfection of hepatocytes with human ApoE4 (h-ApoE4) under control of hepatocyte specific protein arginase-1(Arg1) promotor [19, 20] was achieved by microinjection through hepatic portal vein (Fig. 2B). Following this procedure hepatocytes selectively express h-ApoE4 as shown in Fig. 2C; mice injected with lentivirus without vector served as negative controls. To characterise transport of h-ApoE4, liver and serum exosomes were extracted (Fig. 2D). Protein levels of h-ApoE4 in both exosomal pools were significantly increased in old compared to adult mice (*p* < 0.0001). Adult mice in thyroidectomy group similarly demonstrated increased h-ApoE4 levels in exosomes extracted from liver (*p* = 0.0013) and serum (*p* < 0.0001) (Fig. 2E). Two weeks after injection of h-ApoE4 co-expression vectors the choroid plexus was immunostained for h-ApoE4 and AQP1 (the latter was used to label choroid plexus epithelial cells). The immunoreactivity for liver-originated h-ApoE4 was readily detected in the choroid plexus of old mice, but was rarely observed in adult mice (Fig. 2F). The ApoE4 was detected in choroid plexus epithelial cells thus indicating transcellular transport. Significant amounts of liver-derived h-ApoE4 ended up in cortex and hippocampus of old and thyroidectomized animals (Fig. 2G). Immunofluorescence intensity of h-ApoE4 in cortex and hippocampus was significantly increased in old and thyroidectomized animals as compared with adult group (*p* < 0.0001), whereas no significant difference between adult and sham group has been detected (Fig. 2H).

### Levothyroxine decreases h-ApoE4 in exosomes of old ApoE4-KI mice

**Fig. 3:**
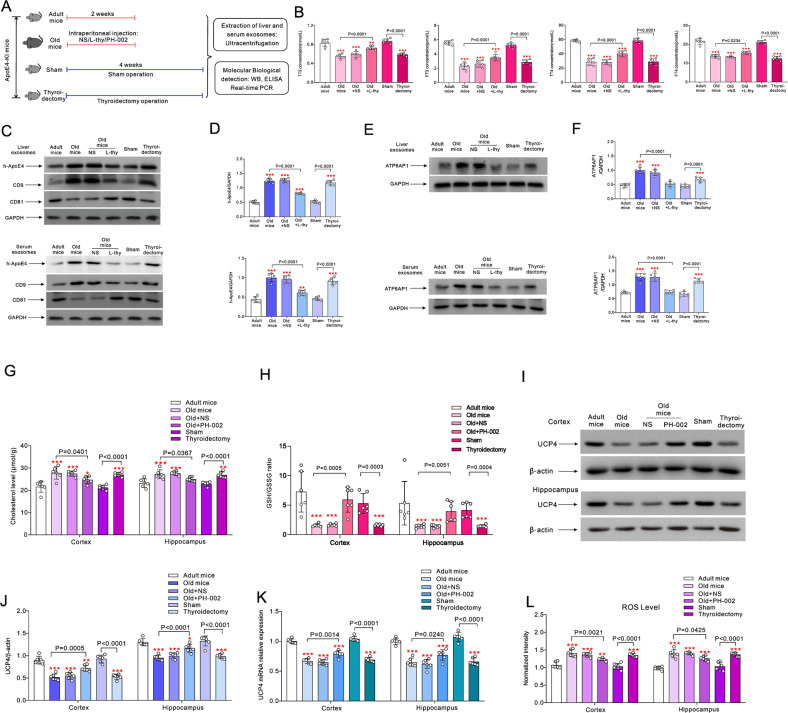
The effects of L-thy and ApoE4 inhibitor on the exosome transport of h-ApoE4 and cerebral oxidative stress in ApoE4-KI mice. **A** Old mice were randomly intraperitoneally injected with normal saline (NS), 20 μg/kg/day L-thy or 200 mg/kg/day ApoE4 inhibitor PH-002 for 14 days; 2 months old mice randomly underwent thyroidectomy or sham surgery. Four weeks after surgery measurements were made. **B** TT3, FT3, TT4 and FT4 values are plotted as mean ± SD, *n* = 6. One-way ANOVA for comparisons including more than two groups; unpaired two-tailed *t*-test for two-group comparisons. As comparison with adult group, *p < 0.05, **p < 0.01, ***p < 0.001. **C** Representative Western blots of h-ApoE4 in the exosomes extracted from liver and serum, CD9 and CD81 were used as the specific markers of exosomes. **D** Expression ratio of h-ApoE4 and GAPDH are plotted as mean ± SD, *n* = 6. **E** Representative Western blots of ATP6AP1 in the exosomes extracted from liver and serum. **F** Ratio of ATP6AP1 normalised to GAPDH is plotted as mean ± SD, *n* = 6. One-way ANOVA for comparisons including more than two groups; unpaired two-tailed *t*-test for two-group comparisons. As comparison with adult group, *p < 0.05, **p < 0.01, ***p < 0.001. **G**, **H** Cholesterol and the ratio of GSH/GSSG in cortex and hippocampus measured by ELISA are plotted as mean ± SD, *n* = 6. **I** Representative Western blots of UCP4 in cortex and in hippocampus. **J** Ratio of UCP4 to β-actin is plotted as mean ± SD, *n* = 6. **K** mRNA expression of UCP4 measured by RT-PCR in cortex and in hippocampus, the relative ratio of UCP4 and GAPDH was normalised to adult group and plotted as mean ± SD, *n* = 6. **L** Mitochondrial ROS level in cortex and hippocampus are plotted as mean ± SD, *n* = 6. One-way ANOVA for comparisons including more than two groups; unpaired two-tailed *t*-test for two-group comparisons. As comparison with adult group, *p < 0.05, **p < 0.01, ***p < 0.001.

We used h-ApoE4 knock-in (ApoE4-KI) mice to characterise exosomal ApoE4 transport in association with age-dependent THs decline. Adult, old, sham and thyroidectomy male ApoE4-KI mice groups were set, as shown in Fig. 3A. Levels of TT3, FT3, TT4 and FT4 were decreased in old (*p* < 0.0001) and thyroidectomized mice (*p* < 0.0001). Administration of levothyroxine (L-thy, 20 μg/kg/day, intraperitoneal injection; i.p.) for 14 days effectively increased THs levels in old mice, as compared with old group (Fig. 3B). Liver and serum exosomal h-ApoE4 levels were significantly increased in old and thyroidectomized mice as compared with adult group (*p* < 0.0001), and there was no significant difference in h-ApoE4 levels between adult and sham groups. Injection of L-thy effectively reduced h-ApoE4 content of liver and serum exosomes compared with untreated old mice (*p* < 0.0001) (Fig. 3C, D). The expression of the exosomal marker CD9 and CD81 were measured simultaneously to control for exosomal purity. Exosomal ATP6AP1 was significantly higher in old and thyroidectomy groups (*p* < 0.0001). Being by injection of L-thy injection significantly decreased (*p* < 0.0001) ATP6AP1 levels in exsosomes from both liver and serum (Fig. 3E, F). Correlation between the h-ApoE4 levels and THs is shown in Supplementary Fig. 2. Similar to observations on human subjects, exosomal h-ApoE4 content demonstrated inverse correlation with THs levels in ApoE4-KI mice (Supplementary Fig. 2A, B).

### Ageing increases cerebral accumulation of ApoE4 and triggers elevation of ROS

In cortex and hippocampus of ApoE4-KI mice, cholesterol levels were significantly increased in both old and thyroidectomized animals when compared to adult controls (*p* < 0.0001). Intraperiotoneal injection of ApoE4 inhibitor PH-002 [21] at 200 mg/kg/day for 14 days, reduced elevated cholesterol in both cortex (*p* = 0.0401) and hippocampus (*p* = 0.0367) of old mice (Fig. 3G). In mitochondria, the ratio of glutathione (GSH) and GSSG (oxidized glutathione) was significantly reduced in the cortex and hippocampus of old and thyroidectomized mice (*p* < 0.0001). Injection of PH-002 recovered reduced ratio of GSH/GSSG in the cortex (*p* = 0.0005) and hippocampus (*p* = 0.0052) of old animals (Fig. 3H). Mitochondrial uncoupling protein UCP4 is expressed in the brain and can be regulated by cholesterol in diabetic rats [22]. The UCP4 acts as a cationic carrier protein in the mitochondrial inner membrane that reduces reactive oxygen species (ROS) production by reducing electron leakage in the respiratory chain [23]. As compared with adult group, the protein expression of UCP4 decreased in the cortex and hippocampus of old and thyroidectomized mice (*p* < 0.0001) (Fig. 3I, J). Similarly, UCP4 mRNA level was downregulated in old and thyroidectomized mice as compared with adult group (*p* < 0.0001) (Fig. 3K). Injection of PH-002 increased protein and mRNA expression of UCP4 in cortex (*p* = 0.0005 and *p* = 0.0014) and hippocampus (*p* < 0.0001; *p* = 0.0240) as compared with old mice group (Fig. 3I–K). Mitochondrial production of ROS in cortex and hippocampus was increased in old and thyroidectomized mice (*p* < 0.0001); this increase was effectively suppressed by PH-002 (*p* = 0.0021 for cortex and *p* = 0.0425 for hippocampus) (Fig. 3L).

### High liver-derived ApoE4 activates NLRP3 inflammasome

**Fig. 4:**
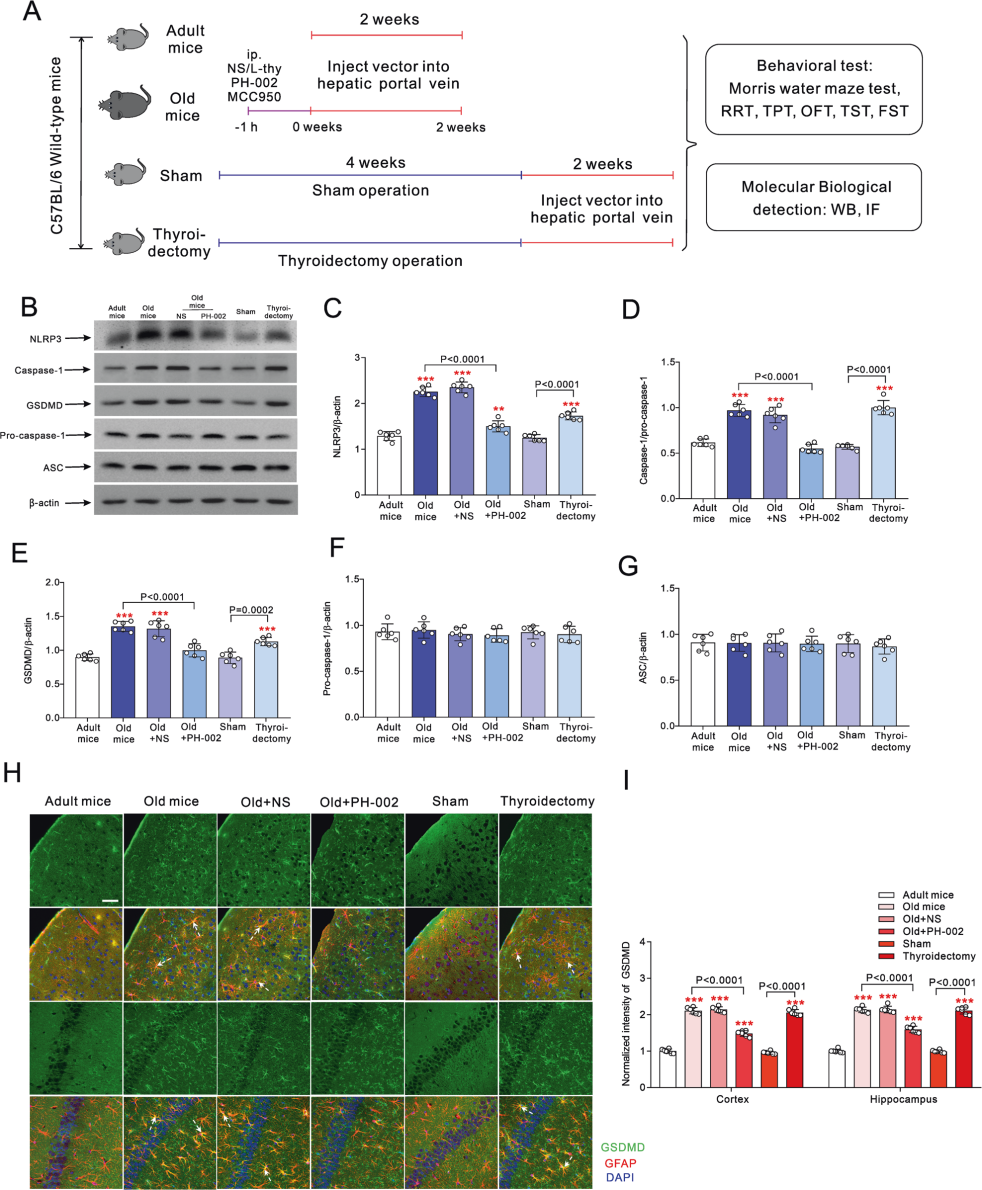
Activation of NLRP3 inflammasome induced by liver-derived ApoE4. **A** Experimental design: 1 h before the hepatic portal vein injection mice in old group were randomly intraperitoneally injected with normal saline (NS), 20 μg/kg/day L-thy, 200 mg/kg/day ApoE4 inhibitor PH-002, lasting for 2 weeks. Two months old mice randomly underwent thyroidectomy or sham surgery. Four weeks after surgery mice were injected with lenti-Arg1-ApoE4-eGFP vector through hepatic portal veins for 2 weeks. **B** Representative Western blots of NLRP3, caspase-1, GSDMD, pro-caspase-1 and ASC collected from the proteins in cortex and hippocampus. **C**–**G** Expression ratios of NLRP3 **C**, caspase-1 **D**, GSDMD **E**, pro-caspase-1, **F** and ASC **G** normalised to β-actin are plotted as mean ± SD, *n* = 6. **H** Immunofluorescence images of GSDMD (green) co-stained with GFAP (red) and DAPI (blue) in cortex (above two rows) and hippocampus (lower two rows). Scale bar = 50 μm. **I** Immunofluorescence intensity of GSDMD normalised to adult group shown as mean ± SD, *n* = 6. One-way ANOVA for comparisons including more than two groups; unpaired two-tailed *t*-test for two-group comparisons. As comparison with adult group, *p < 0.05, **p < 0.01, ***p < 0.001.

Effects of liver-derived ApoE4 on the neural cells and behavioural performance were studied in adult, old, sham and thyreoidectomy animals (Fig. 4A). In addition, L-thy, ApoE4 inhibitor PH-002 or the selective inhibitor of NLRP3 inflammasome (MCC950) were intraperitoneally injected 1 h before the injection of lenti-Arg1-ApoE4-eGFP vector in old mice. Two weeks after liver vector transfection, protein expressions of NLRP3, caspase-1 and gasdermin D (GSDMD) were significantly increased in the cortex and hippocampus of the old and thyroidectomized mice (*p* < 0.0001), while injection of PH-002 downregulated their expression (*p* < 0.0001) (Fig. 4B–E). Expression of associated pro-caspase-1 and ASC (apoptosis-associated speck-like protein containing a caspase-activation and recruitment domain (CARD)) was not affected by ageing or dysfunctional thyroid hormone secretion (Fig. 4F, G). Pyroptosis related protein GSDMD was co-localised with astrocytic marker GFAP in cortex and in hippocampus (Fig. 4H). Immunofluorescence intensity of GSDMD staining increased in old and thyroidectomized mice (*p* < 0.0001). Injection of PH-002 significantly suppressed the GSDMD immunoreactivity of aged animals (*p* < 0.0001) (Fig. 4I). Images of brain tissues co-labelled with antibodies against GSDMD, microglial marker Iba1 and neuronal marker NeuN are presented in Supplementary Fig. 3A. Intensity of GSDMD labelling in microglia and neurons increased in old or thyroidectomized mice while PH-002 reversed this increase (Supplementary Fig. 3B).

### Liver-derived ApoE4 causes behavioural impairments

**Fig. 5:**
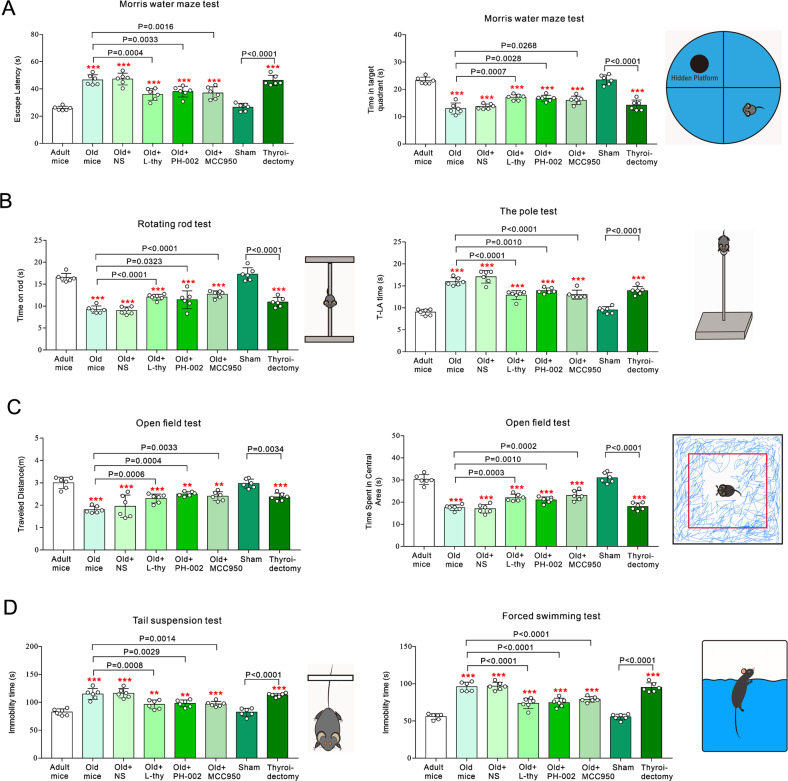
Behavioural impairments induced by liver-derived ApoE4. **A** Cognitive function in Morris water maze test. Time of escape latency and time in target quadrant were recorded and shown as mean ± SD, *n* = 6. **B** Motor function in rotarod and pole tests. Time on rod and T-LA time are shown as mean ± SD, *n* = 6. **C** Anxiety-like behaviours in open field test. Total travel distance and time spent in the central area were shown as mean ± SD, *n* = 6. **D** Depressive-like behaviours in tail suspension and forced swimming tests. Immobility time are shown as mean ± SD, *n* = 6. One-way ANOVA for comparisons including more than two groups; unpaired two-tailed *t*-test for two-group comparisons. Compared to adult group, *p < 0.05, **p < 0.01, ***p < 0.001.

In Morris water maze the time of escape latency was significantly increased in old or thyroidectomized wild type mice with liver transfection of lenti-Arg1-ApoE4-eGFP vector as compared with adult mice (*p* < 0.0001). This increase was reversed by injections of 20 μg/kg/day L-thy, 200 mg/kg/day PH-002 or 50 mg/kg/day MCC950 (the selective inhibitor of NLRP3 inflammasome). Similarly, L-thy, PH-002 and MCC950 alleviated decreased time in target quadrant observed in old or thyroidectomized mice (Fig. 5A). Rotarod test and pole test were applied to characterise motor control in ApoE4-KI mice (Fig. 5B). The time on rod for old or thyroidectomized mice was significantly decreased (*p* < 0.0001): while the T-LA time (turn downward from the top of pole (T-turn time) and descend to the floor) was significantly longer in old and thyreoidectomied animals (*p* < 0.0001). Administration of L-thy, PH-002 or MCC950 significantly improved motor function in old mice (Fig. 5B). In the open field test the travelled distance and the time spent in central area were decreased in old or thyroidectomiz/ed mice (*p* < 0.0001); both parameters were normalised by L-thy, PH-002 or MCC950 (Fig. 5C). Tail suspension and forced swimming tests showed longer immobility times in old and thyroidectomised mice (*p* < 0.0001); treatments with L-thy, PH-002 or MCC950 shortened the immobility time in old mice (Fig. 5D).

## Discussion

**Fig. 6:**
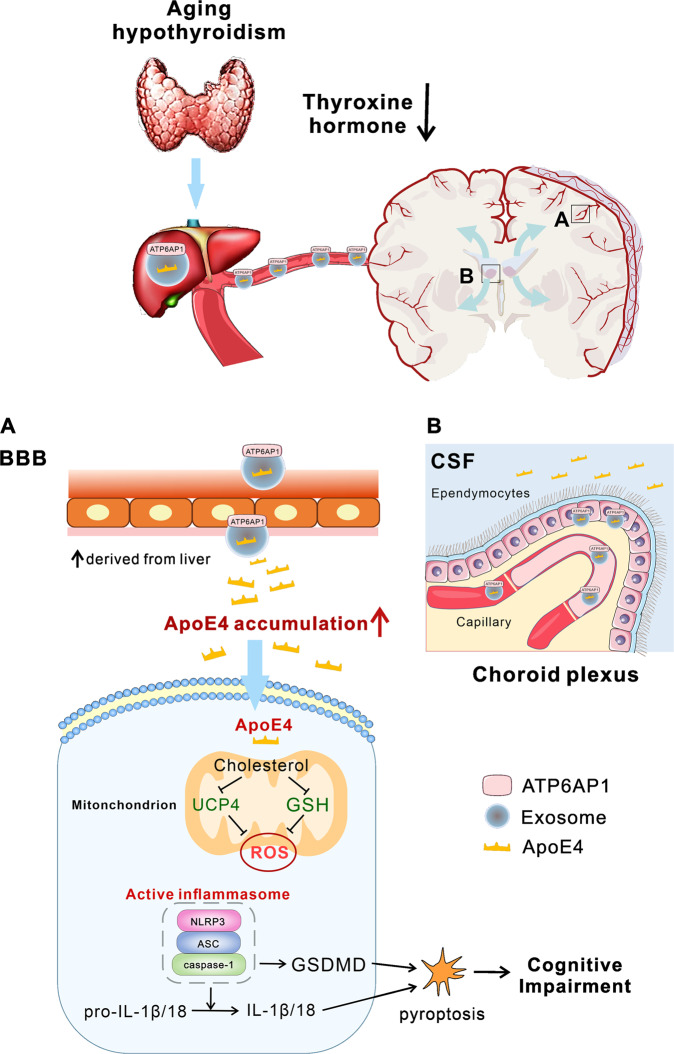
Ageing related hypothyroidism promotes exosomal transport of liver-derived ApoE4 to the brain, thus activating NLRP3 inflammasome and cause neurological impairments. Ageing-associated hypothyroidism promotes liver-derived ApoE4 exosomal transport. ApoE4-laden exosomes exosomes to cross BBB entering the brain **A**, liver-derived ApoE4-reach exosomes also enter CSF in choroid plexus **B**. Both processes result in the accumulation of ApoE4 in the brain. Elevated ApoE4 increases the cholesterol level in neural cells, which suppresses mitochondrial content of GSH and UCP4 thus leading to an increase in ROS. Increased ROS activates NLRP3 inflammasome triggering pyroptosis, that contributes to neurocognitive decline and depression.

The ApoE4 allele is an acknowledged risk factor of age-associated cognitive impairments and AD. Here we demonstrate that in old animals ApoE4 produced in the liver is transported, with exosomes, to the brain. Increase of the brain-targeted exosomal transport is linked to the age-dependent decline in THs secretion and is possibly mediated by an increased expression of ATP6AP1 component of the V-ATPase in the exosomes extracted from liver and serum. Exosomes carrying ApoE4 cross the blood–brain barrier, entering the cerebrospinal fluid through choroid plexus by transcellular route (Fig. 6A). Subsequently ApoE4 is distributed throughout the cerebral parenchyma, resulting in the accumulation of ApoE4 in brain (Fig. 6B). In neural cells, elevated ApoE4 increases intracellular cholesterol level that accelerates accumulation of ROS through suppressing GSH and UCP4 mitochondrial content. Elevated ROS trigger activation of NLRP3 inflammasome and instigates pyroptosis of neural cells, which contribute to cognition, motor and mood impairments (Fig. 6). Our clinical studies support the above statements, as the levels of ApoE4 in serum exosomes is significantly higher in patients with AD-related dementia, which is associating with the decline in THs and cognitive abilities.

In postmenopausal women, low cognitive functions are associated with the presence of ApoE4 allele and lower levels of FT3 and TT3, although thyroid-stimulating hormone (TSH) levels are increased [24]. Lower serum TSH levels in combination with ApoE4 allele were shown to impair memory and cognitive performance of elder Koreans [25]. We studied 60 carriers of ApoE4 allele including 32 male and 28 female subjects, with the gender ratio 1.14. We did not detect any gender-dependent differences in exosomal ApoE4 content. At the same time levels of ApoE4 in exosomes extracted from serum and liver demonstrate inverse correlation with cognitive function and levels of TT3, FT3, TT4 and FT4. Similar inverse correlation between the levels of four THs and exosomal ApoE4 content was found in ApoE4-KI mice (Supplementary Fig. 2). Furthermore, ApoE4 levels in serum exosomes were higher in cognitively compromised AD patients when compared to healthy old subjects. Thus, increased level of exosomal ApoE4 may be used as biological marker for the early diagnosis of AD.

We subsequently found that the exosomes mediate the transport of ApoE4 from the liver to the brain. Decreased THs secretion in ageing affects expression of ATP6AP1 in exosomes, which in turn modifies exosomal transport. The activity of V-ATPase is regulated by reversible separation of the V_1_ and V_O_ regions, while the ATP6AP1 is the main component of the mammalian V_O_ region [26]. We discovered that increase in ATP6AP1 associated with ageing may promote exosomal transport by increasing exosomal secretion from hepatocyte (Fig. 3E, F). The V_0_-ATPase was shown to regulate the formation of multivesicular bodies, exosomal content of Hedgehog-related proteins and apical secretion of exosomes in *Caenorhabditis elegans* [27].

Intracellularly accumulated ApoE4 competes with ApoE2 for high-density lipoprotein (HDL) to block the extracellular transportation of cholesterol [28]. In addition, accumulation of ApoE4 into neural cells may impair the mitochondrial function [29, 30]. We demonstrated that the age-related accumulation of h-ApoE4 significantly increased cholesterol and decreased GSH in cortex and hippocampus. We also found age-dependent upregulation of UCP4 which may contribute to the production of mitochondrial ROS. Expression of UCP4 is decreased in the patients of AD [31], while an intronic variant of neuronal mitochondrial UCP4 gene increases the risk of late-onset AD and frontotemporal dementia [32]. In the rat type 2 diabetic model, the lipid-lowering drug bezafibrate increases the expression of UCPs mRNA in liver by lowering cholesterol level [22]. At the same time, accumulation of cholesterol in mitochondria can inhibit the transport of GSH, resulting in the depletion of mitochondrial GSH, increased ROS formation and oxidative stress [33, 34]. Enhanced production of ROS in cortex and hippocampus triggers activation of NLRP3 inflammasome [35]. NLRP3 inflammasome is a multiprotein complex of the adaptor protein ASC and inflammatory caspase-1. Activation of NLRP3 inflammasome activates caspase-1 and accelerates maturation and release of pro-inflammatory factors, such as IL-1β and IL-18 [35]. During this process, GSDMD, a physiological substrate of the canonical inflammasome pathway is recruited to the NLRP3 inflammasome causing pyroptosis and release of mature IL-1β/18 through plasma membrane pores [36, 37]. Here, we showed that liver-derived ApoE4 triggers cognitive impairments, motor and depressive- or anxiety-like behaviours by activating NLRP3 inflammasome.

In AD patients, cognitive and motor deficits are frequently associated with neuropsychiatric manifestations [38]. Neuronal loss in the prefrontal cortex (PFC) and hippocampus [39, 40], as well as an increased release of pro-inflammatory cytokines [41], link AD with mood disorders. We demonstrate that increased exosomal transport of ApoE4 from the liver to the brain associated with the age-related TH, affects cognitive functions and evokes depressive- and anxiety-like behaviours. These changes occur together with ApoE4-induced activation of NLRP3 inflammasome and pyroptosis of neural cells. In conclusion, age-associated TH decline increases ApoE4 transport into the brain thus contributing to the high incidence of AD-related dementia and neuronal impairments in ApoE4 allele carriers.

## Materials and methods

### Materials

Primary antibody of GSDMD (sc-393656), ASC (sc-365611), caspase-1 (sc-56036), AQP1 (sc-25287) and ATP6AP1 (sc-515607) were purchased from Santa Cruz Biotechnology (Santa Cruz, CA, USA). Primary antibody of pro-caspase-1 (ab179515), GFAP (ab48050), UCP4 (ab183886), secondary antibody Alexa Fluor 555 donkey anti-rabbit (ab150074), Alexa Fluor 647 goat anti-chicken (ab150171), and TT3 ELISA kit (ab108685), FT3 ELISA kit (ab108663), TT4 ELISA kit (ab178664), FT4 ELISA kit (ab108686) were purchased from Abcam (Cambridge, MA, USA). Primary antibody of ApoE4 (MA5-16146), Arg1 (PA5-85267), MAP2 (PA1-16751), NeuN (PA5-78639), NLPR3 (768319), CD9 (MA5-31980), CD81 (MA5-32333), secondary antibody Alexa Fluor 488 donkey anti-rat (A21208) and electrochemiluminescence (ECL) detection reagents (#32132) were purchased from Thermo Fisher Scientific (Waltham, MA, USA). Primary antibody of β-actin (E021020) and secondary antibody HRP-labelled goat anti-mouse (E030110) and HRP-labelled goat anti rabbit (E030120) were purchased from Earthox (Millbrae, CA, USA). Primary antibody of GAPDH (60004-1-Ig) was purchased from Proteintech (Chicago, USA). Iba1 antibody used for immunofluorescence was purchased from Wako Chemicals (USA). The ROS assay kit (BB-470532) was purchased from BestBio (Shanghai, China). The GSH assay kit (CS0260-1KT), cholesterol quantitation kit (MAK043-1KT), PH-002 (SML0461) and levothyroxine (T-073) were purchased from Sigma-Aldrich (St. Louis, Missouri, USA). Co-expression lentivirus vectors of Arg1-ApoE4 (human)-eGFP (NM_007482-linker-NM_0000041) were designed and organised from Genechem (Shanghai, China). The quantitative PCR (RR820A and RR047A) was purchased from TaKaRa Bio (Kusatsu, Japanese). MCC950 (CP-456773) was purchased from MedChem Express (Shanghai, China). Mitochondria extraction Kit (G006-1-1) was purchased from Jiancheng Bioengineering (Nanjing, China).

### Clinical studies

This study was performed on 60 ApoE4 heterozygous participants, including 15 healthy adult subjects, 15 healthy old subjects and 30 patients with clinical diagnosis of AD-related dementia. Participants were chosen from 93 healthy adult subjects (20–50 years old), 95 healthy old subjects (>50 years old) and 83 diagnosed AD-related dementia patients (>50 years old) by DNA sequencing of the extracted venous blood, which were carried out by TaKaRa Biotechnology (Dalian, China). All participants have junior high school or above education background. Healthy adult and old subjects were selected from age-matched individuals, who did not have neurodegenerative diseases, stroke and brain tumour. AD-related dementia patients were diagnosed with AD per the criteria of the National Institute on ageing-Alzheimer’s Association and excluded vascular dementia [42, 43]. This study was authorised and approved by Medical Ethics Committee of China Medical University (No. [2020]059) and registered on Chinese Clinical Trial Registry (registration number: ChICTR2000029731).

### Mini-Mental State Examination (MMSE) scores

MMSE is the most commonly used dementia screening tool that can be accomplished in the relatively short time of 5–10 min. The MMSE consists of 30 questions with a maximum score of 30. The MMSE tests the following seven cognitive domains: orientation in time and place, memory registration and recall, attention and calculation, and language. The MMSE score of 26 or higher is representative of both cognitive and physical health. Scores under 25 indicate cognitive impairment [44].

### Montreal Cognitive Assessment (MoCA) scores

MoCA is a screening method to detect cognitive function [45]. Performing MoCA takes about 10–15 min. There are 12 items for cognitive domains; memory is tested by a short-term memory recall task (5 points); visuospatial ability is tested using a clock-drawing test (CDT; 3 points) and a 3-dimensional cube copy (1 point); executive function is tested using a trail-making test, part B (TMT-B; 1 point), a phonemic fluency task (1 point), and a 2-item verbal abstraction task (2 points); attention, concentration, and working memory is tested using a sustained attention task (1 point), a serial subtraction task (3 points), and digits forward and backward tasks (1 point each); language is tested using a 3-item confrontation naming task with low familiarity animals (lion, camel, rhinoceros; 3 points) and repetition of 2 syntactically complex sentences (2 points); orientation in time and place was also tested (6 points). The maximum score of MoCA is 30, and higher scores indicate better cognitive function. The score of MoCA ≥ 26 is representative of both cognitive and psychological healthy, score from 11 to 21 shows the cognitive disorders [45].

### Animals

Wild type C57BL/6 mice and h-ApoE4 replacement transgenic mice (B6.129-Apoe^tm3(APOE*4)Mae^Ldlr^tm1(LDLR)Mae^/J) were purchased from the Jackson Laboratory (Bar Harbor, ME, USA). In this study, only male mice were used. Adult mice (3 months) were randomly divided into three groups: adult group, sham and thyroidectomy group; old mice (18 months) were randomly divided into five groups: old group, normal saline injection group, levothyroxine injection group, ApoE4 inhibitor (PH-002) injection group and NLRP3 inflammasome inhibitor MCC950 injection group. The method of randomisation was the random number table. The mice were raised in standard housing conditions (22 ± 1°C; light/dark cycle of 12/12 h), with water and food available *ad libitum*. All experiments were performed in accordance with the US National Institutes of Health Guide for the Care and Use of Laboratory Animals (NIH Publication No. 8023) and its 1978 revision, and all experimental protocols were approved by the Institutional Animal Care and Use Committee of China Medical University, No. [2021]117.

### Thyroidectomy

Adult mice were anesthetised with ketamine (80 mg/kg, i.p.) and xylazine (10 mg/kg, i.p.), and placed on their backs to expose the neck area. A midline skin incision was made along the length of the neck. Underlying tissues were cleared and the salivary glands were retracted laterally. Two halves of the sternohyoid muscle were separated and retracted laterally. Thyroid muscle was separated from the thyroid gland lobes and retracted along with the sternohyoid muscle. A midline cut was made in the isthmus and the thyroid glands were excised bilaterally. Extreme care was taken not to damage the laryngeal nerve. Sham group underwent the same surgical procedures without removal of the thyroid gland. Mice were monitored closely for at least 1 week after the surgery for complications and animals were used for experimentation 4 weeks later [46].

### Microinjection into hepatic portal vein

Wild type C57BL/6 mice were anesthetized according to the above methods and fixed on the operating table. Abdominal hair has removed with chemical depilatory cream. Using a sterile scalpel, a 2.5 cm incision was made through the skin between the positive and sagittal planes on the left side of the mouse. Subsequent using sterile scissors and tweezers, 2.5 cm incision was cut in the peritoneum. A 10 × 10 cm gauze was soaked with normal saline and placed it in the left incision. Holding the middle side of the incision with tweezers, the large and small intestine were carefully pulled out with a sterile cotton swab and placed on the gauze to expose the portal vein. Internal organs were covered in the saline-soaked gauze to maintain internal moisture and sterility. Injection of the co-expression lentivirus vectors of Arg1-ApoE4 (human)-eGFP, was done through the needle inserted at about 10 mm below the liver and 3–5 mm into the portal vein with an angle <5° and bevel facing up; 5 μl containing lentivirus at 4 × 10^7^ TU was slowly injected. The negative control group was injected only with lentivirus without the target vector. Mice were individually housed after full recovery from anaesthesia. After 2 weeks, the mice were used for the experiments [47].

### Serum exosome extraction

In clinical settings, the blood samples of healthy subjects and patients were collected at 10 ml drawn from the median cubital vein. For taking blood samples from animals, each mouse was anesthetised and blood was collected by inserting a capillary glass tube into the medial cantus of the orbit while gently pushing and rotating it forward. We used EP tube to collect venous blood from seven mice as one sample. After the collection of the blood samples, serum was separated from the blood by centrifugation at 3400 x g for 10 min. Serum samples were centrifuged at 800 x g for 5 min, then 2000 × g for 10 min and 13,500 x g for 20 min. The supernatant was filtered using a 0.2 μm syringe filter (Corning or Whatman, USA). The filtrated serum was purified by ultracentrifugation (Beckman Coulter Optima L-90K) at 198,000 x g for 4 h to collect exosomes. Exosome pellets were resuspended in 200 μl phosphate buffer saline (PBS) and stored at −80 °C [48]. Transmission electron microscopy and nanoparticle tracking analysis assays were used to identify exosomes (Supplementary Fig. 1A, B).

### Liver exosome extraction

After anaesthesia as above, the mice were perfused through myocardium with Hank’s buffer. The liver tissue was taken out and placed in a clean beaker. The livers of five mice were collected as one sample. The liver was chopped, digested with 0.125% trypsin for 20 min, and then poured into the glass homogeniser. The homogenised liver tissues were filtered with a 40 μm screen. Then, the filtrated solution was transferred to a 15 ml centrifuge tube, and was centrifuged at 3000 x g for 10 min. Liver samples were centrifuged at 800 x g for 5 min, then 2000 x g for 10 min and 12,000 x g for 20 min. The supernatant was filtered using a 0.2 μm syringe filter. The filtrated serum was purified by ultracentrifugation (Beckman Coulter Optima L-90K) at 150,000 x g for 4 h to collect exosomes. Exosome pellets were resuspended in 200 μl PBS and stored at −80 °C [49, 50].

### Immunofluorescence (IF)

Anesthetised mice were perfused through myocardium with 4% paraformaldehyde (PFA) for 15 min. The brain or liver tissues were cut into 60 μm slices. Brain or liver slices were blocked and permeabilized by incubation for 1 h with 5% donkey serum solution containing 0.5% Triton^TM^ X-100 in PBS. Primary antibodies against ApoE4 or GSDMD were used at a 1:100 dilution, against Arg-1, aquaporins 1 (AQP1), glial fibrillary acidic protein (GFAP), microtubule associate protein 2 (MAP2), NeuN and Iba1 used at 1:200 dilution. The solution of primary and secondary antibodies was the dilution of 0.1% Triton^TM^ X-100 in PBS. Nuclei were stained with marker 4', 6'-diamidino-2-phenylindole (DAPI) at 1:1000 dilution. The incubation with the primary antibodies lasted overnight at 4 °C and then donkey anti-rat or anti-rabbit Alexa Fluor 488/555, or goat anti-chicken Alexa Fluor 647 conjugated secondary antibody for 2 h at room temperature. Images were captured using a confocal scanning microscope (DMi8, Leica, Wetzlar, Germany) [51]. As described previously [52], the intensity of the immunofluorescence was co-localized with DAPI or cell-specific markers GFAP, NeuN, or Iba1. Background intensities per image were measured in cell-free parenchyma in the same field of view and subtracted from the total immunofluorescence intensities. The intensity of h-ApoE4 or GSDMD immunofluorescence from the different groups was normalized to the intensity of adult mice group (control).

### Western blotting

For quantifying expressions of h-ApoE4, ATP6AP1, NLRP3, pro-caspase-1, caspase-1, GSDMD, ASC, UCP4, β-actin and GAPDH, the protein content was determined by the Bradford method [53], then the samples containing 100 μg of protein were added to 10% SDS-polyacrylamide gel electrophoresis. After electrophoretic separation and the gels were transferred to PVDF membranes, the samples were blocked by 5% skimmed milk powder for 1 h, and membranes were incubated overnight with the primary antibodies, specific to either human-specific ApoE4 at 1:1000 dilution, ATP6AP1 at 1:500 dilution, NLRP3 at 1:1000 dilution, pro-caspase-1 at 1:1000 dilution, caspase-1 at 1:1000 dilution, GSDMD at 1:1000 dilution, ASC at 1:1000 dilution, UCP4 at 1:1000 dilution, β-actin at 1:5000 dilution and GAPDH at 1:3000 dilution. After washing, specific binding was detected by horseradish peroxidase-conjugated secondary antibodies. Staining was visualised by ECL detection reagents and was analysed with an Electrophoresis Gel Imaging Analysis System (MF-ChemiBIS 3.2, DNR Bio-Imaging Systems, Israel). Band density was measured with Window AlphaEaseTM FC 32-bit software [54].

### Real-time PCR

To measure the mRNA of UCP4, the RNA of cortex and hippocampus was extracted by Trizol reagent (Invitrogen, Carlsbad, CA, USA). Total RNA was reverse transcribed to cDNA by using a reverse transcription reagent kit (Takara, Otsu, Shiga, Japan) in a Robo-cycler thermocycler. Real-time PCR was performed with the LightCycler 480 SYBR Green I Master kit (4887352001, purchased from Roche) and the products were detected using a LightCyler 480 instrument (Roche, Mannheim, Germany). The sequences of Real-time PCR primers used for mRNA quantification were as follows: 5′-ATTGCGATTTCGTGGTGTACAT-3′ and 5′-CCATATTCACCAGTGCTGCTCTT-3′ for mice UCP4 and 5′-TGGTGCCAAAAGGGTCATCTCC-3′ and 5′-GCCAGCCCCAGCATCAAAGGTG-3′ for mice GAPDH. Relative genomic expression was calculated by the 2^−ΔΔCt^ method [55, 56].

### Mitochondria extraction

Mitochondria isolation and fractionation was conducted by using the mitochondria extraction kit, according to the manufacturer’s protocol. Fresh collected tissues of cortex and hippocampus were homogenised with an all-glass Dounce homogeniser in cold lysis buffer. Then, the homogenates were centrifugated at 800 × g, 4 °C for 5 min. After centrifugation, the supernatant was slowly transferred to a new pre-cooled tube, which pre-added with 500 μl extraction reagent A. To centrifuge the sample tubes at 15,000 × g, 4 °C for 10 min, the supernatant was obtained as cytoplasm extract. The sediment was suspended with 200 μl wash buffer, and then centrifuged at 15,000 × g, 4 °C for 10 min again. To dispose the supernatant, the sediment was resuspended with 100 μl storage buffer and kept under −80 °C.

### ROS level detection

To assay the ROS level, a commercial ROS assay kit (BB-470532, BestBio, Shanghai, China) was used and operated as the protocols. The ROS assay kit based on the oxidant-sensitive fluorescent dye 2′,7′-dichlorodihydrofluorescin diacetate (DCFH-DA). The tissues of cortex and hippocampus were incubated with a 1 ml serum-free culture medium containing 10 μM of DCFH-DA. After incubation for 30 min at 37 °C, the cells were washed with warm PBS three times to remove the excess dye. Finally, the fluorescence intensity of each well was measured using a fluorescence microplate reader (Infinite M200 Pro, Hombrechtikon, Switzerland). The excitation wavelength was at 488 nm, and the emission peak was examined at 525 nm.

### ELISA assays

The concentrations of TT3, FT3, TT4 and FT4 in serum were determined using a commercial ELISA kit based on the manufacturer’s instructions. The levels of cholesterol and GSH in tissue samples from the cortex and hippocampus were measured using a commercial ELISA kit based on the manufacturer’s instructions. Cytokine levels in samples were determined in duplicate following assay instructions provided by the manufacturers. The optical density (OD) of each microwell was measured at 450 nm using a microplate reader.

### Behavioural tests

In behavioural tests, mice were randomly assigned to avoid interference from different test with a random number table. All behavioural tests were performed by an investigator blinded to the experimental conditions. Behavioural tests were recorded, stored, and analysed by using the SMART™ tracking software program (Panlab, SL, Barcelona, Spain). The apparatus was cleaned with 25% ethanol solution after each test.

### Morris Maze test

Morris water maze test is to check a spatial learning and memory. The mice were trained over five consecutive days in four different quadrants. The mice were trained to locate and escape onto the platform during this period. A different starting position for each mouse was used each time. Animals that failed to find the location within 90 s were guided to the platform and were allowed to remain on it for 20 s. The platform position was fixed throughout the test. In the place navigation test, the escape latencies for each mouse per day were calculated. In the memory retention session, a single test was conducted in the pool, in which the hidden platform was removed; a 1-min probe trial was conducted to measure the time spent in the target quadrant by each mouse [52].

### Rotating rod test

The rotating rod test is a test for assessing motion ability in mice. The individual mouse was placed on the rotating bar, which was set to a rotation speed at 20 rpm during the test. The time spent on the rotating bar was recorded as the latent period. The latency before falling was recorded using a stop-watch, with a maximum of 90 s. Time of staying on rotating rod indicates motion balance ability [57].

### Pole test

The pole test is also a test for assessing motion ability in mice. Each mouse was paced head-upward on the top of a vertical rough-surfaced pole (diameter 1 cm; height 55 cm). The turn downward from the top of pole (T-turn time) and the descend to the floor (T-LA) time was recorded [58].

### Open field test

The open field test is an anxiety-based test. The mice were placed in the centre square of an open field box (60 × 60 × 40 cm) divided into nine squares, and behaviours were recorded for 6 min. The parameters used for analysis included the total travel distance and the time spent in the central area [57].

### Forced swimming test

The forced swimming test is a despair-based behavioural test. Each mouse was trained to swim for 15 min before the formal measurement. Then the trained mouse was put into a glass cylinder that contained 30 cm deep water (25 ± 1 °C) and left for 6 min. The immobility time was recorded during the last 4 min that followed 2 min of habituation [59].

### Tail suspension test

The tail suspension test is also a despair-based behavioural test. Mice were suspended by their tails at 20 cm from ground for 6 min. The time of immobility was recorded to calculate percentage. The immobility time in tail suspension test reflects despair-like behaviour [58].

### Statistics and reproducibility

For statistical analysis, we used one-way analysis of variance (ANOVA) followed by a Tukey’s or Dunnett’s post hoc multiple comparison test for unequal replications using GraphPad Prism 8 software (GraphPad Software Inc., La Jolla, CA) and SPSS 24 software (International Business Machines Corp., NY, USA). One-way ANOVA for comparisons including more than two groups; unpaired two-tailed *t*-test for two-group comparisons. All statistical data in the text are presented as the mean ± SD; the value of significance was set at *p* < 0.05.

## Supplementary information

Supplementary figure legends

Supplementary Figure 1

Supplementary Figure 2

Supplementary Figure 3

Western blots original pictures

checklist

## Data Availability

The full and uncropped pictures of western blots are available in supplementary material. The data that support the findings of this study are available from the corresponding author Baoman Li upon reasonable request.
