# Supplementary material for: Ageing related thyroid deficiency increases brain-targeted transport of liver-derived ApoE4-laden exosomes leading to cognitive impairment
Source: Cell Death Dis. 2022 Apr 25;13(4):406. doi: 10.1038/s41419-022-04858-x (PMC9039072; doi:10.1038/s41419-022-04858-x)
Supplement: Supplementary file 1 — Supplementary figure legends [file 41419_2022_4858_MOESM1_ESM.docx]

**Supplementary Figure Legends**

**Supplementary Figure 1. Transmission electron microscopy and nanoparticle tracking analysis for exosomes.**

(A) Transmission electron microscopy (magnification, 80000x) was used to identify exosomes. Scale bar 200 nm

(B) Nanoparticle tracking analysis assays was used to measure exosomal sizes distribution.

**Supplementary Figure 2. Correlation analysis of TH levels and ApoE4 of exosomes.**

Correlation between the levels of TH (TT3, FT3, TT4 and FT4) and h-ApoE4 of exosomes extracted from liver (A) and serum (B), respectively. TH levels are taken from Fig. 3B and h-ApoE4 levels from Fig. 3C; the comparison of TH and h-ApoE4 were from the same mice.

**Supplementary Figure 3. Immunofluorescence of GSDMD in microglia and neurones.**

Experimental design: 1 hour before the hepatic portal vein injection mice in old group were randomly intraperitoneal injected with normal saline (NS) or 200 mg/kg/day ApoE4 inhibitor PH-002, lasting for 2 weeks. 2 months old mice randomly underwent thyroidectomy or sham surgery. 4 weeks after surgery mice were injected with lenti-Arg1-ApoE4-eGFP vector through hepatic portal veins for 2 weeks.

(A) Immunofluorescence images of GSDMD (green) co-stained with nucleus marker DAPI (blue), Iba-1 (red; upper) or neuronal marker NeuN (red; lower) in cortex and hippocampus. Scale bar = 50 μm.

(B) Immunofluorescence intensities of GSDMD in old microglia and neurones normalised to the adult group are shown as mean ± SD, n = 6.

One-way ANOVA for comparisons including more than two groups; unpaired two-tailed t-test for two group comparisons. As comparison with adult group, *p<0.05, **p<0.01, ***p<0.001.
