# Supplementary figures and images for: Ageing related thyroid deficiency increases brain-targeted transport of liver-derived ApoE4-laden exosomes leading to cognitive impairment
Source: Cell Death Dis. 2022 Apr 25;13(4):406. doi: 10.1038/s41419-022-04858-x (PMC9039072; doi:10.1038/s41419-022-04858-x)

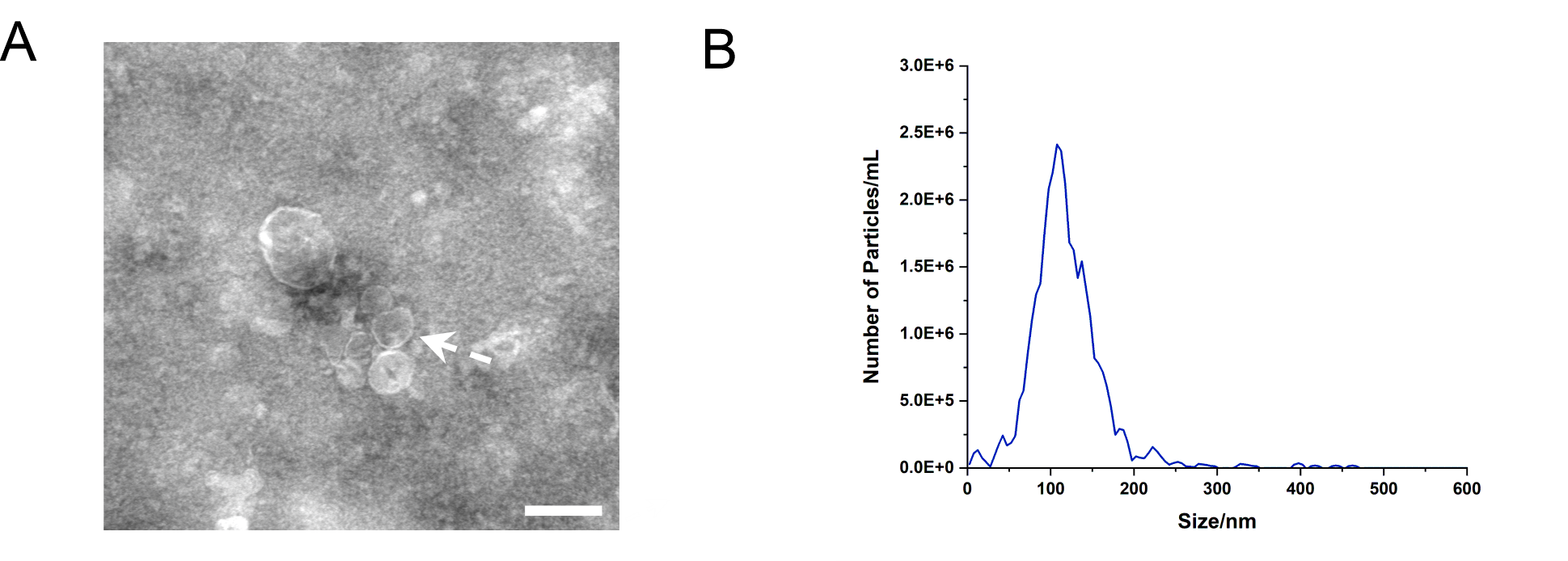

Supplement: Supplementary file 2 — Supplementary Figure 1 [file 41419_2022_4858_MOESM2_ESM.tif]

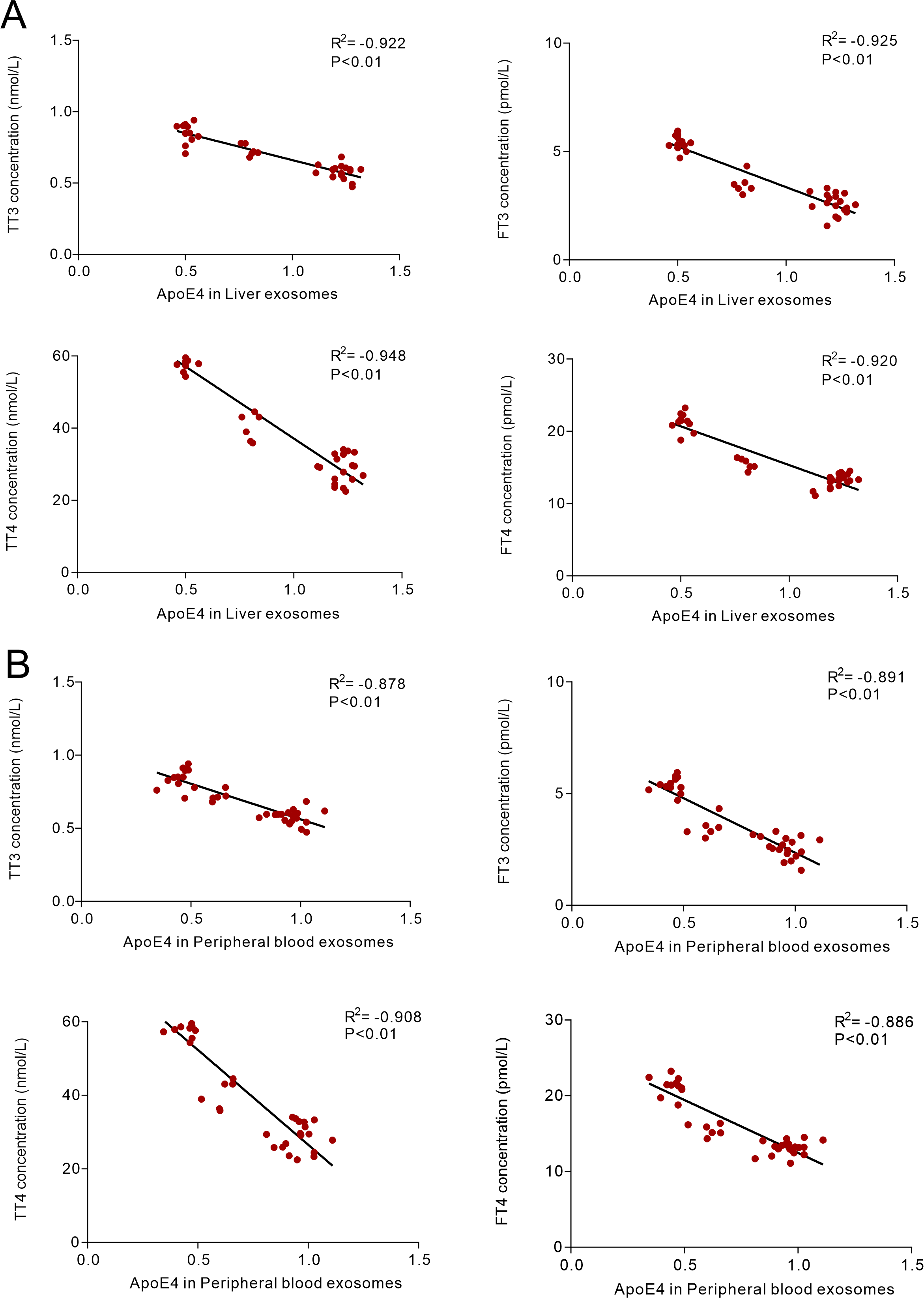

Supplement: Supplementary file 3 — Supplementary Figure 2 [file 41419_2022_4858_MOESM3_ESM.tif]

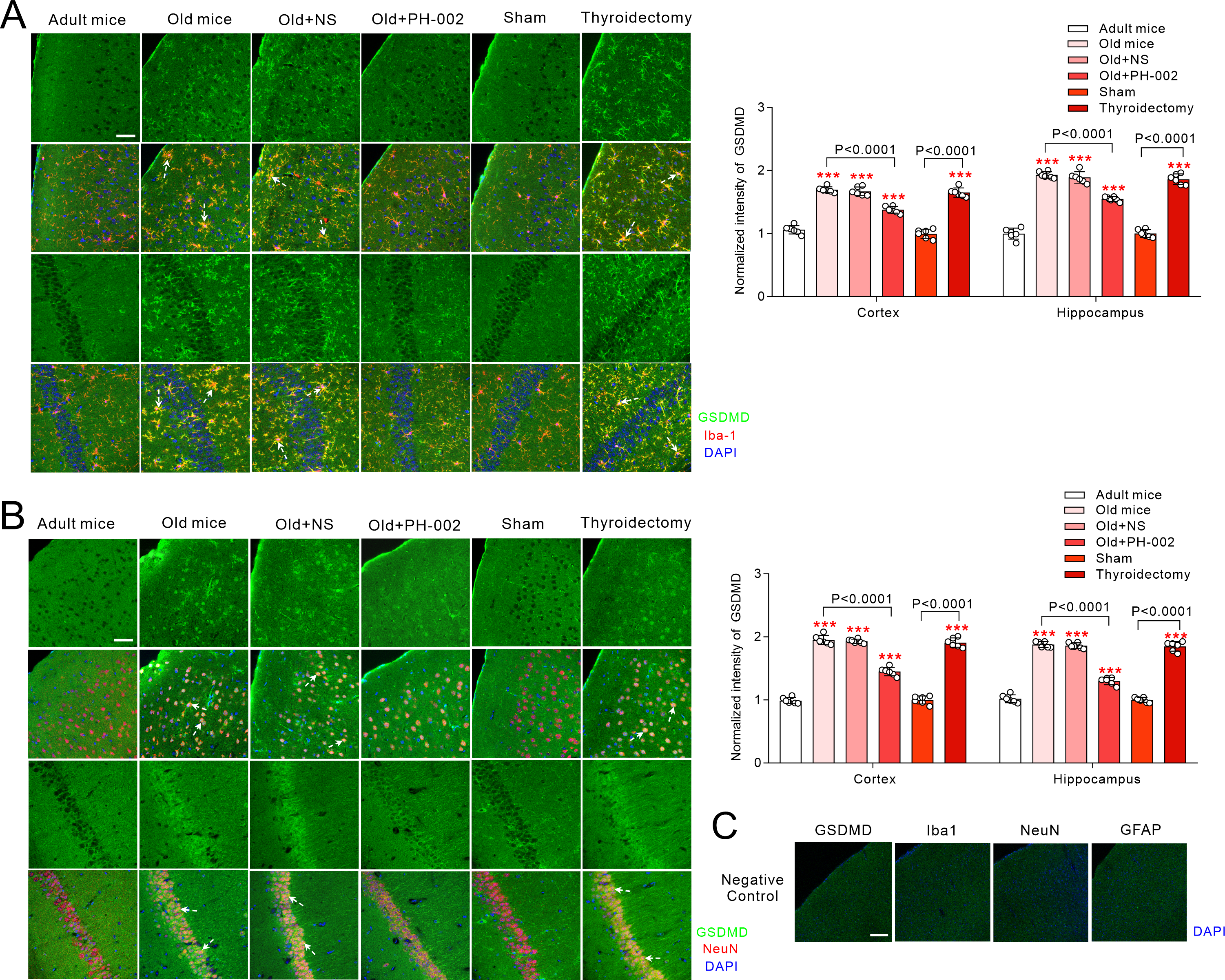

Supplement: Supplementary file 4 — Supplementary Figure 3 [file 41419_2022_4858_MOESM4_ESM.tif]

Figure 1


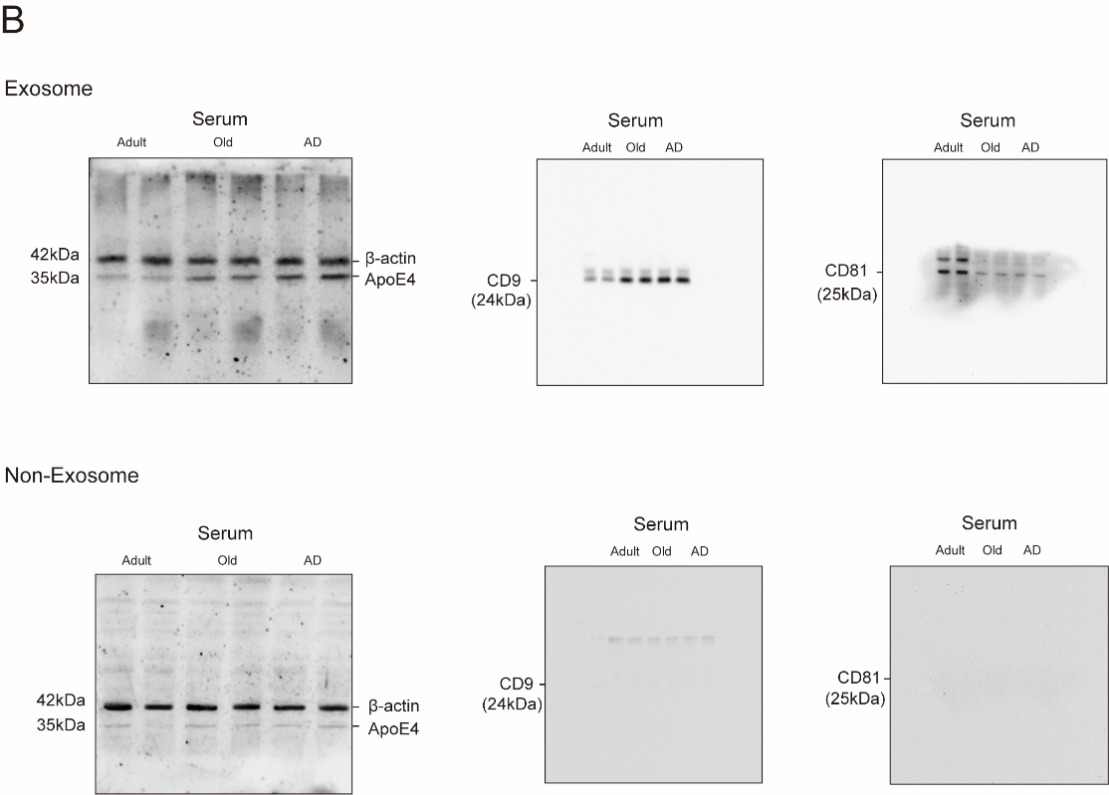


Figure 2D


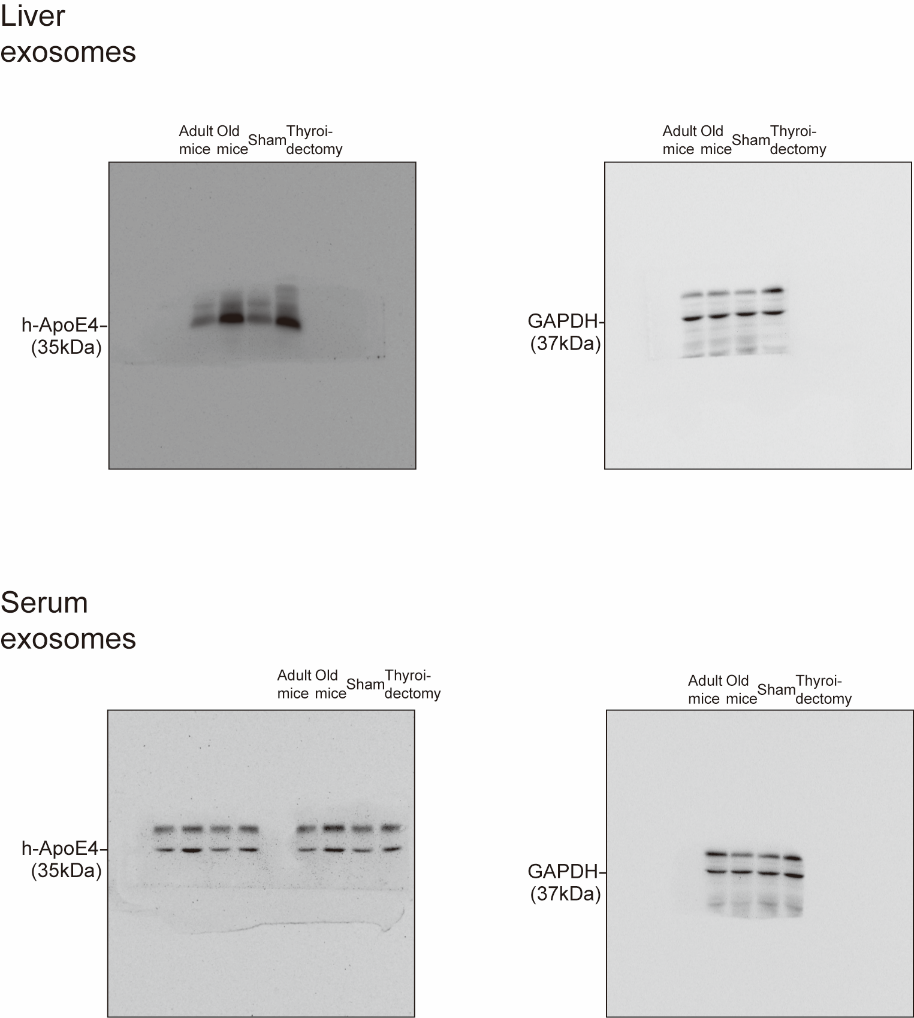


Figure 3


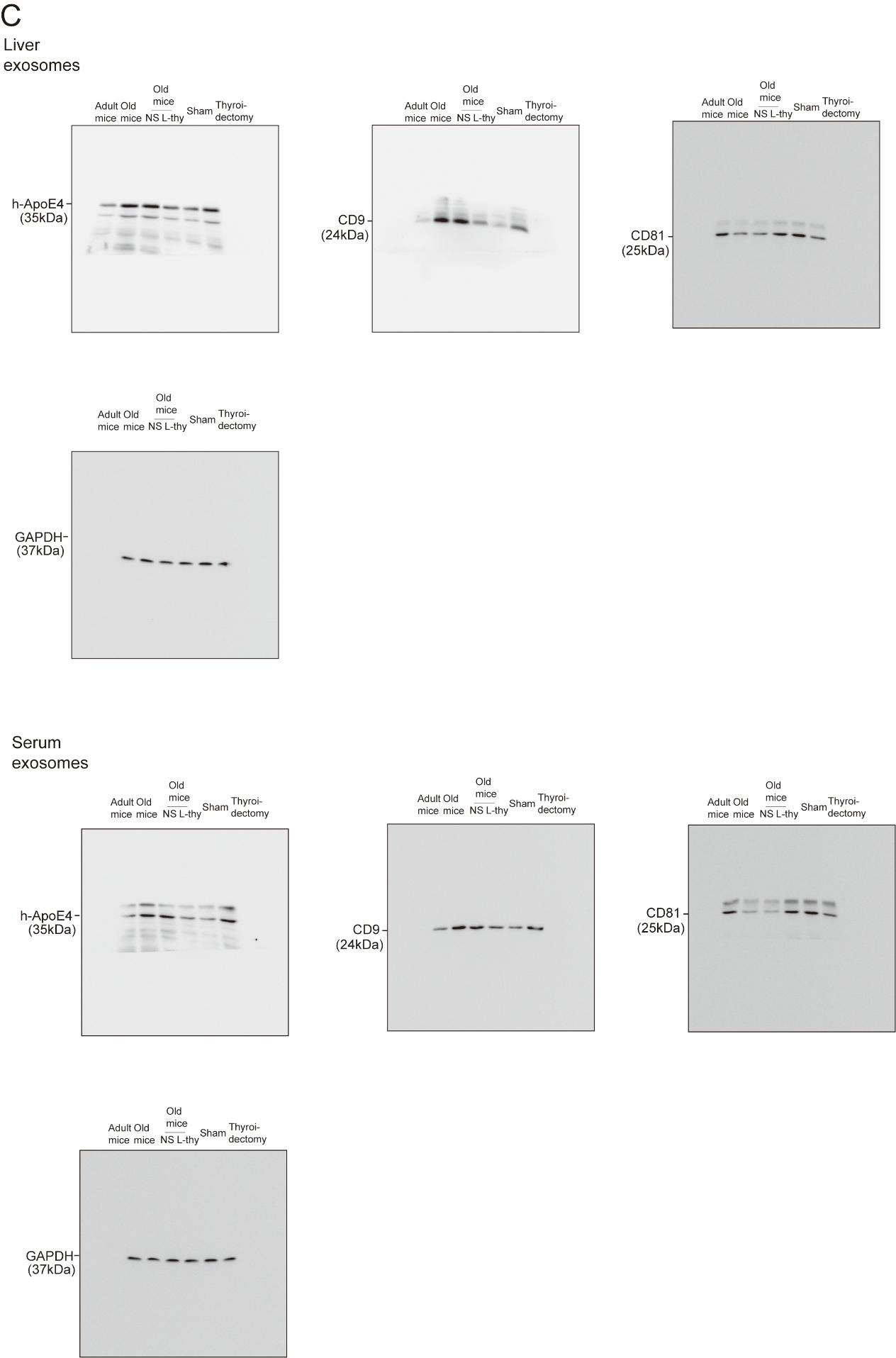


Figure 3


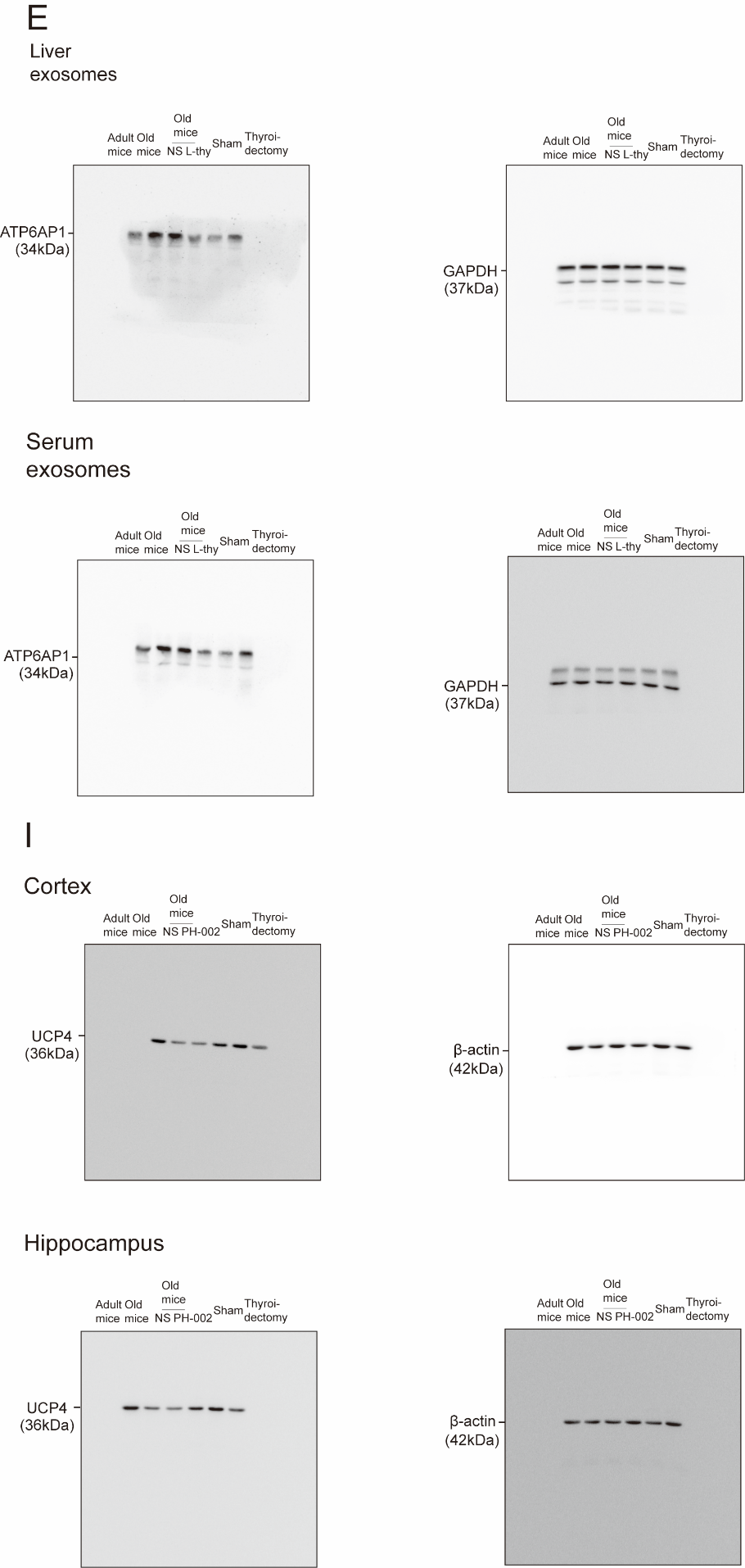


Figure 4B


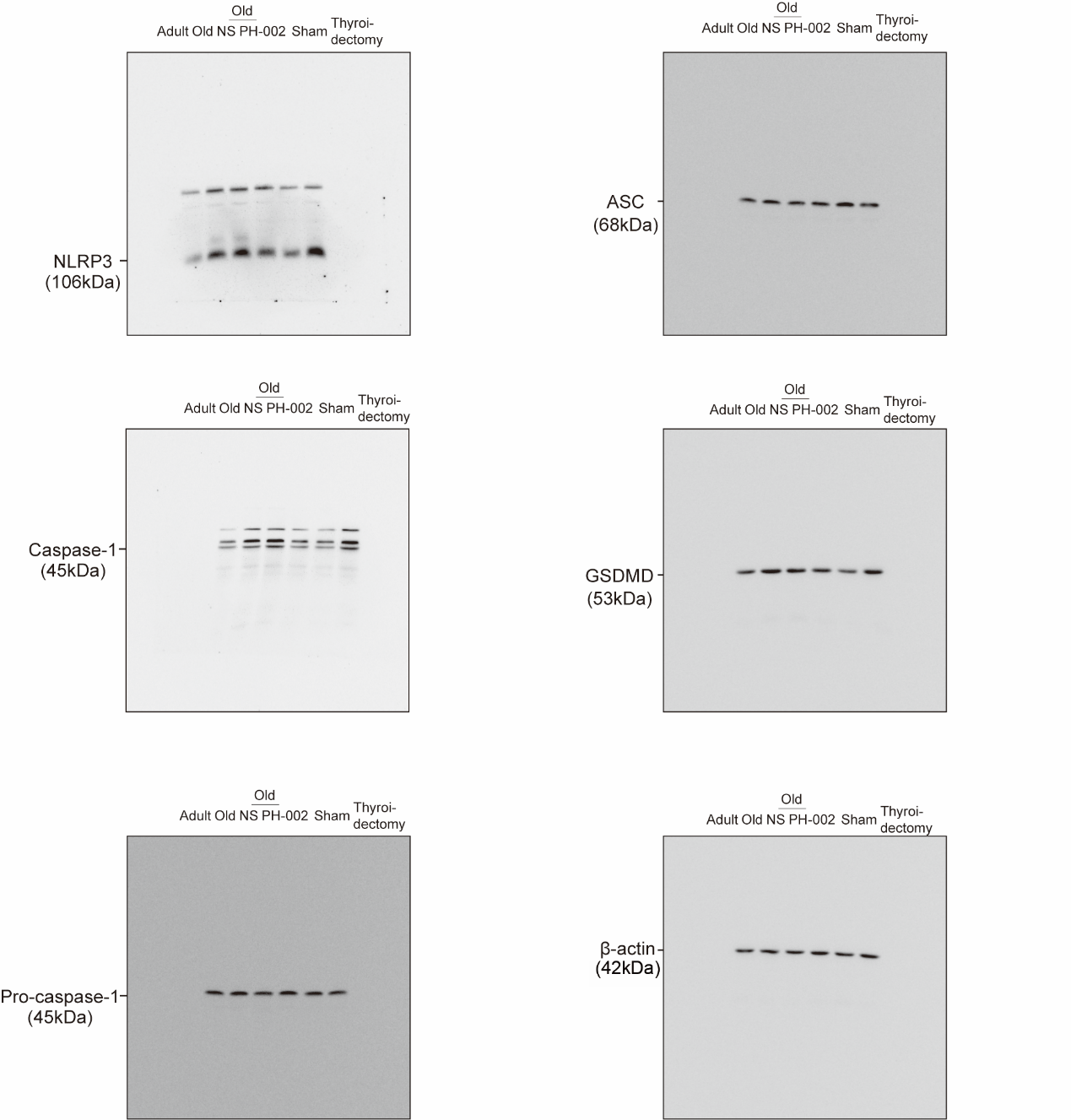

Supplement: Supplementary file 5 — Western blots original pictures [file 41419_2022_4858_MOESM5_ESM.docx]
